# Effects of Watercress (*Nasturtium nasturtium*) extract on selected immunological parameters of rainbow trout (*Oncorhynchus mykiss*)

**Published:** 2012-05-10

**Authors:** M.S. Asadi, A.R. Mirvaghefei, M.A. Nematollahi, M. Banaee, K. Ahmadi

**Affiliations:** 1 *Department of Fishery, Natural Resource Faculty, University of Tehran, Karaj, Iran*; 2 *Department of Aquaculture, Natural Resource and Environmental Faculty, Behbahan University of Technology, Iran*; 3 *Young Researchers Club, Tehran North Branch, Islamic Azad University, Tehran, Iran*

**Keywords:** Hematological parameters, Immunological parameters, *Nasturtium nasturtium*, Rainbow trout, Watercress extract

## Abstract

Watercress (*Nasturtium nasturtium*) is a medical plant containing diverse chemically-active substances with biological properties. The present study was conducted to investigate the immunomodulatory effects of watercress extract on immunological and hematological parameters of rainbow trout (*Oncorhynchus mykiss*). Fish were fed for 21 days with diet supplemented with 0.1% and 1% of watercress extract per 1 kg food and with a normal diet as control. Hematological parameters such as red blood cells (RBC) and white blood cells (WBC), hematocrit (Hct), hemoglobin (Hb), RBC index like mean corpuscular volume (MCV), mean corpuscular hemoglobin (MCH) and mean corpuscular hemoglobin concentration (MCHC) as well as immunological parameters such as peroxidase, lysozyme and complement activities, total protein, albumin and globulin levels were measured after 21 days of watercress extract treatment. The results indicated that oral administration of 1 % watercress extract in fish may enhance some hematological and immunological parameters including Hb and MCHC, lysozyme and complement activities, total protein and globulin levels, compared to the controls after 21 days of experimental period. In conclusion, on the basis of these results, oral administration of watercress extract may be useful to improve fish’s immune system.

## Introduction

Many scientists believe that the word consumption of medical plants is growing rapidly (Aliyu *et al.*, 2007). Although the application of antibiotics and chemicals can be effective in controlling pathogens in aquatic animal farms, the residues of these compounds in meat may be a threat to the health of consumers (Biswas *et al.*, 2010).

Recently, several efforts have been made to replace chemical drugs by herbal medicine in aquaculture industry in many countries (Dügenci *et al.*, 2003; Citarasu *et al.*, 2006; Obaroh and Achionye-Nzeh, 2011). In fact, most research has focused on the role of plant extracts in stimulating the immune system in fish comparing with bacterial, parasitic and fungal agents (Sivaram *et al.*, 2004; Rao and Chakrabarti, 2005; Citarasu *et al.*, 2006; Rao *et al.*, 2006; Divyagnaneswari *et al.*, 2007; Sarkar *et al.*, 2011).

Nevertheless, there are concerns and doubts about the use of herbal medicine and their derivatives as alternative drugs (Hashemi and Davoodi, 2012). These studies did not investigate the impact of herbal drugs on health status of the fish. Therefore, our knowledge about usage of commercial herbal medicine in aquaculture is very limited. Thus, research on the effects of these compounds before administration is necessary. Watercress (*Nasturtium nasturtium*, Cruciferae family) is a perennial plant which growths in clear, cold water and is found in ditches and streams everywhere.

Watercress, which is cultivated for its pungent leaves which are used in cooking especially in soups, garnishes and salads, is one of the most important herbal medicines used in traditional treatment of some diseases such as diabetes (Shahrokhi *et al.*, 2009), oxidative stress (Yazdanparast *et al.*, 2008), asthma (Goda *et al.*, 1999), scorch (Abu-Zinadah, 2008) and immune depression (Sonnenbichler *et al.*, 1986). Raw watercress leaves are used as salad greens, or can be steamed and consumed as a normal processed vegetable.

Watercress is a valuable source of vitamins and a good detoxifying herb. This plant contains a relatively large amount of vitamins B1, B2, C and pro-vitamin A, folic acid, glucosinolates, iodine, iron, protein, and especially calcium and sulphur compounds, which influence its characteristic odor, but also adds to its nutritional benefits (Chung *et al.*, 1992; Rose *et al.*, 2000; Palaniswamy *et al.*, 2003). The active constituents of watercress (*Nasturtium nasturtium*) extract may strengthen or stimulate the immune response by interacting with various parameters of the immune system.

According to published research findings, watercress is the richest source of glucosinolates, which can be hydrolyzed to produce phenethyl isothiocyanate. Isothiocyanates can prevent carcinogen activation through the inhibition of phase I enzymes, such as cytochrome P 450s (Conaway *et al.*, 1996) and through triggering phase II enzymes such as quinine reductase (quinine-acceptor), oxidoreductase, glutathione -S- transferase (GST) and glucuronosyltransferases, resulting ultimately in the excretion of potential carcinogens (Wallig *et al.*, 1998; Bianchet *et al.*, 1999). In addition, administration of watercress extract can inhibit of fibrosis (Abu-Zinadah, 2008), inhibit of inflammation (Goda *et al.*, 1999), inhibit of P450 activity (Chung *et al.*, 1992) and have antioxidant properties and inhibit of lipid peroxidation (Wallig *et al.*, 1998; Bianchet *et al.*, 1999).

Based on scientific findings, Kolawole *et al*. (2011) stated that one way to distinguish the appropriate or inappropriate prescription of medical plants is the assessment of their effects on hematological and biochemical parameters in experimental animals. Although the effect of oral administration of watercress on hematological and immunological parameters in experimental animals was previously studied, there is not any information about the impact of using watercress extract on different parameters of immune system of fish.

Measuring the alterations in innate immunity and non-specific immune parameters of treated fish with herbal derivatives is one way to evaluate the effects of herbal drugs on immune system of fish. Therefore, the aim of the present study is to assay the effect of watercress oral supplement on some non-specific immune parameters of rainbow trout.

## Materials and Methods

### Fish and experimental procedure

Juvenile rainbow trout (*Oncorhynchus mykiss*, average weight 96±10 g, and average length 14±2 cm) were purchased from a private farm (Rainbow trout farm, Kamiaran, Iran.) and were transferred to another private farm (Gavshan village, Kamiaran, Iran). After transport, fish were randomly parceled in nine closed water recirculating systems (1000 L) for at least two weeks to acclimate to the laboratory conditions (15±2º C; pH,7.4±0.2; 212±3 mg per CaCO_3_; 6±0.5 mg/L DO, 20% water exchange rate/day) prior to experiments. During acclimation, fish were fed with commercial diets (Behparvar Co. Iran) at 2% of their body weight twice a day.

During experimental period, the fish were fed for 21 days with 0.1 and 1% watercress extract per 1 kg food, and commercial diet only as controls, to examine the effect on immune parameters. The watercress was collected from the bank of Gav River (Gavshan village, Kamiaran, Iran) and the extracts were prepared according to procedure described by Banaee *et al*. (2011). In this method, watercress leaves were extracted with chloroform–methanol (2,1 V/V) in a warming blender. This extract was dried over anhydrous sodium sulfate, and the solvent was removed from the filtrate under reduced pressure at 45°C.

Watercress extracts were then mixed with commercial diets (Behparvar Co. Iran; Table 1) to achieve doses of 0.1 and 1 % per 1 kg of fish feed. During the experimental period, fish were fed with enriched commercial trout pellets with 0 (control), 0.1, and 1 % of watercress extract per one kg of food at 2% of their body weight twice a day. During the trials, fish were observed for appetite. All fish were deprived of food for 24 hours before weighing and sampling. The following growth parameters were measured at the end of the 21-day trial.

**Table 1:** Nutrient composition of the commercial diets (Behparvar Co. Iran)

Nutrient composition	Reference diet
Dry material	92.54
Metabolize energy (Kcal/g)	350.24
Crud protein	40.22
Ether extract (lipid)	10.49
Ash	7.86
Crude fiber	5.79
Carbohydrate	27.36


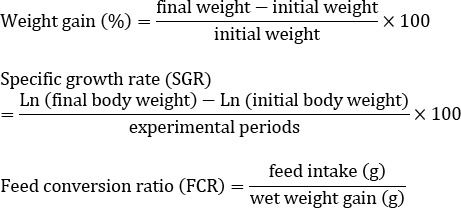


Immunostimulatory activity was evaluated after the 21^st^ day of the initial experiments; 12 fish per treatment were captured and anesthetized under aquatic solution of clove powder (1,5000). Fish from each experimental and control groups were bled from the dorsal aorta into sterilized glass vials at 4 °C containing the anticoagulant ethylene glycol tetra-acetic acid (EDTA) at 1%.

The blood was centrifuged for 15 min at 4000 g, 4ºC. Plasma were immediately stored at -80ºC until biochemistry and immunostimulatory activity analysis.

### Hematological parameters

The blood was immediately used to determine the number of red blood cells (RBC) and white blood cells (WBC) by means of a haemocytometer slide at a magnification of 400X. Thus, blood was diluted to 10^-2^ and 10^-3^ in phosphate-buffered saline (PBS) at pH 7.2 (Sarder *et al.*, 2001). Hematocrit (Hct) was determined by the microhematocrit method described by Brown (1988). Hemoglobin (Hb) concentration was conducted by using the cyanohaemoglobin method (Azizoglu and Cengizler, 1996). Red cell indices, mean corpuscular volume (MCV, μm3/cell), mean corpuscular hemoglobin (MCH, pg/cell), and mean corpuscular hemoglobin concentration (MCHC, g/l) were calculated from RBC, Ht, and Hb according to Banaee *et al*. (2008) as follows:


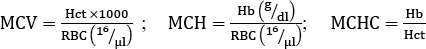


### Alternative complement activity

Alternative complement activity (ACH50) was evaluated following the procedure of Yano (1992) by using rabbit red blood cells (RaRBC). Briefly, RaRBC were washed and adjusted to 2 × 10^8^ cell/ml in EDTA-magnesium-gelatin veronal buffer (0.01 M). Precisely 100 μl of the RaRBC suspension was lysed with 3.4 ml of distilled water and the absorbance of the haemolysate was measured at 414 nm against distilled water to acquire the 100% lysis value.

The test plasma was appropriately diluted, and different volumes ranging from 0.1 to 0.25 ml were made up to 0.25 ml total volume before being allowed to react with 0.1 ml of RaRBC in test tubes. After incubation at 20ºC for 90 min with occasional shaking, 3.15 ml of a 0.9% (w/v) saline solution was added to each tube with centrifugation at 1600 *xg* for 10 min at 4ºC. The absorbance (A) of supernatant was measured using a spectrophotometer at 414 nm. A lysis curve was obtained by plotting the percentage of haemolysis against the volume of plasma added. The volume of plasma producing 50% haemolysis (ACH50) was determined and the number of ACH50 units/ml was obtained for each fish.

### Lysozyme activity

The turbidimetric assay for lysozyme was carried out according to Lange *et al*. (2001) with minor modifications. Thus, plasma (50 µl) was added to 2 ml of a suspension of *Micrococcus lysodeikticus* (0.2 mg/ml) in a 0.05 M sodium phosphate buffer (pH 6.2). The reaction was carried out at 25ºC and absorbance was measured spectrophotometrically at 570 nm after 0.5 min and 4.5 min. PBS was used as a blank. A unit of lysozyme activity was defined as the sample amount causing a decrease in absorbance of 0.001/min. Lysozyme of sample calibrated using a standard curve determined with hen’s egg white lysozyme (Sigma) in PBS.

### Peroxidaes content

The total peroxidase content in plasma was measured according to the method used by Cuesta *et al*. (2007). Briefly, 10 µl of plasma was diluted in 100 µl of Hank’s balanced salt solution (HBSS) buffer. Then, 50 µl of 20mM 3,3’,5,5’–tetramethylbenzidine hydrochloride and 2.5 mM hydrogen peroxide were added. The color change reaction was stopped after 2 min by adding 50 µl of 2M sulphoric acid and the optic density was read at 450 nm. Standard samples without plasma were also analyzed. The peroxidase activity (unit/ml plasma) was determined defining one unit of peroxidase as that which produces an absorbance change of 1 OD.

### Blood biochemical parameters

Plasma total protein and albumin levels were measured by using the total protein and albumin kit (Parsazma Co. Iran). Globulin levels were calculated by subtracting albumin values from plasma total protein.

### Statistical analysis

Statistical analyses were performed using SPSS (Release 15) software. Data are presented as mean ± SD. All the data were tested for normality (Kolmogorov-Smirnov test). Data were analyzed by one-way of variance analysis (ANOVA). The significant means were compared by Tukey’s test and a p < 0.05 was considered statistically significant.

## Results

No significant differences were observed in the final weight, final length, weight gain, specific growth rate (SGR) and food convert ratio (FCR), among different experimental groups and these groups and the control group (Table 2).

**Table 2:** Changes in final weight, final length, weight gain, specific growth rate (SGR) and food convert ratio (FCR), of rainbow trout fed for 21 days with 0.0, 0.1 and 1% of watercress extract per 1 kg food.

Concentration of watercress extract	Control	0.1% WCE	1% WCE
Final Weight (g)	115.33±13.88^a^	116.89±16.60^a^	116.86±14.37^a^
Final Length (cm)	17.26±1.93^a^	16.89±2.01^a^	17.24±1.76^a^
Weight gain (%)	0.21±0.08^a^	0.22±0.08^a^	0.19±0.06^a^
SGR (%)	0.39±0.13^a^	0.41±0.14^a^	0.36±0.11^a^
FCR	1.25±0.15^a^	1.24±0.16^a^	1.24±0.15^a^

Significant differences between values were characterized by alphabetical symbols (one-way ANOVA, p<0.05). Values represent mean ± S.D. differences when compared with control groups.

For hematological parameters (Table 3), there were no significant differences in RBC and WBC counts, Hct, MCV and MCH values between all of treatments and control groups on the 21^st^ day. Hb concentrations were significantly increased when fish were treated with diets enriched with 1% of watercress extract per 1 kg food on the 21^st^ day when compared to control (*p* < 0.05). MCHC value of fish fed with 1% of watercress extract per 1 kg food was significantly higher than its value in the control group on 21^st^ day (Table 3).

**Table 3:** Erythrocyte (RBC), leukocyte (WBC) counts and hematocrit (Hct) and hemoglobin (Hb), MCV, MCH and MCHC values of rainbow trout fed for 21 days with 0.1 and 1% of watercress extract per 1 kg food.

Hematological Parameters	Concentrations of watercress extract		
	Control	0.1% WCE	1% WCE
RBC (10^6^/µl)	1.26±0.15^a^	1.22±0.17^a^	1.28±0. 20^a^
WBC (10^4^/µl)	19.94±3.24^a^	21.44±4.03^a^	20.61±3.18^a^
Hb (g/dL)	10.47±2.32^a^	11.40±3.01^ab^	12.75±2.87^b^
Hct (%)	44.29±5.21^a^	45.51±3.44^a^	43.67±3.53^a^
MCV (mm^3^,10^-5^)	35.5±3.89^a^	38.1±5.89^a^	34.7±5.47^a^
MCH (pg,10^-5^)	8.44±2.25^a^	9.69±3.34^a^	10.20±2.86^a^
MCHC (%)	23.67±5.35^a^	25.33±7.30^ab^	29.39±6.97^b^

Significant differences between values were characterized by alphabetical symbols (one-way ANOVA, p<0.05). Values represent mean ± S.D. differences when compared with control groups.

No significant change in peroxidase activity in plasma of fish fed with watercress extract was observed when compared with control group during experimental period. ACH50 levels were significantly increased when fish were treated with diets enriched with 1% of watercress extract during experimental period. Generally, lysozyme activity was highest in fish fed with 1% of watercress extract after 21 days (Table 4).

**Table 4:** Changes in peroxidase, total complement (ACH50), lysozyme, and total protein, and albumin, globulin levels in blood of rainbow trout fed for 21 days with 0.0, 0.1 and 1% of watercress extract per 1 kg food.

Biochemical Parameters	Concentrations of watercress extract		
	Control	0.1% WCE	1% WCE
Peroxidase (U/mL)	128.95±17.74^a^	124.39±10.47^a^	122.28±9.23^a^
ACH50 (U/mL)	315.72±25.16^a^	304.95±18.14^a^	372.67±23.59^b^
Lysozyme (U/mL)	116.56±11.45^a^	125±11.25^a^	134.05±9.10^b^
Protein (mg/dL)	5.53±0.73^a^	5.59±0.70^ab^	6.12±0.54^b^
Albumin (mg/dL)	3.61±0.65^a^	3.42±0.58^a^	3.68±0.51^a^
Globulin (mg/dL)	1.92±0.38^a^	2.17±0.36^ab^	2.43±0.55^b^

Significant differences between values were characterized by alphabetical symbols (one-way ANOVA, p<0.05). Values represent mean ± S.D. differences when compared with control groups.

Total protein levels were significantly increased in the fish fed with enriched food by 1% of watercress extract following 21 days of treatment when compared with the control group (*P*<0.05). No significant changes in albumin levels in plasma of fish fed with watercress extract-enriched food when compared with control group (*P*<0.05). Consumption of food containing 1% of watercress extract had a significant effect on globulin concentrations in plasma of experimental fish after 21 days (Table 4).

## Discussion

The present study focused on the immunostimulantory properties of watercress extract containing a relatively large amount of vitamins B1, B2, C and pro-vitamin A, folic acid, glucosinolates, iodine, protein, iron, calcium and sulphur compounds (Chung *et al.*, 1992; Rose *et al.*, 2000; Palaniswamy *et al.*, 2003) on hematological and immunological parameters of rainbow trout (*Oncorhynchus mykiss*).

Although, the results obtained in this study show that oral administration of the watercress extract had no significant effect on growth rate of experimental fish, some recent studies have shown that feeding herbal supplementary food to fish resulted in increased disease resistance and in improved survival and growth rate, which may be attributed to an improvement of immune functions (Christybapita *et al.*, 2007; Divyagnaneswari *et al.*, 2007; Ardó *et al.*, 2008; Cheng *et al.*, 2008).

Hematology, including erythrocyte count, haemoglobin concentration, haematocrit and leucocyte count, has provided valuable information for fishery biologists in the assessment of fish health (Banaee *et al.*, 2008). Although our results suggest that while oral administration of 1% watercress extract for at least 21 days may increase hemoglobin content (Hb) and MCHC values, no significant alternations were observed in the number of erythrocytes and leukocytes as well as hematocrit, MCV and MCH values. In other words, oral administration of watercress extract may concentrate hemoglobin in red blood cells of fish. Increase in hemoglobin content (Hb), hematocrit, and numbers of leucocytes and thrombocyte were reported in Nile tilapia (Shalaby *et al.*, 2006) and hybrid tilapia (Ndong and Fall, 2011) fed with diet enriched by garlic. These results were in agreement with previous findings where feeding with other herbal supplementary food led to an increase in hemoglobin levels (Martins *et al.*, 2002; Ji *et al.*, 2007).

Lysozymes are a family of enzymes with antibacterial activity characterized by the ability to damage the cell wall of bacteria. So, significant increase in lysozyme activity in plasma of fish fed for 21 days with diet enriched with 1% of watercress extract may indicate an increase the fish’s immune system defense against bacterial agents. Yet, feeding with 0.1% of watercress extract did not reveal a significant difference in lysozyme activity relative to the controls. Ardó *et al*. (2008) reported increase in lysozyme activities of Nile tilapia (*Oreochromis niloticus*) fed for seven days with Chinese herbs *Astragalus membranaceus* and *Lonicera japonica*. Furthermore, according to scholar’s reports, the use of *Astragalus radix* (Yin *et al.*, 2006), *Eclipta alba* (Christybapita *et al.*, 2007), *Ganoderma lucidum* (Yin *et al.*, 2009) incorporated into the fish diet and fed for 14 to 60 days led to a significant increase in the lysozyme activity.

Complement includes over 20 different plasma proteins that are produced by a variety of cells including, hepatocytes, macrophages and gut epithelial cells. Some complement proteins bind to immunoglobulins or to membrane components of cells. The complement system is an essential and effective part of the innate immune system. It can rapidly distinguish and opsonize bacteria for phagocytosis by specialized phagocytes or destroy them directly by membrane disorder (Rooijakkers and van Strijp, 2007).

The enhancement of complement activity (ACH50) in plasma of fish fed with diet enriched with 1% of watercress extract may indicate the improvement of the fish immune system ability during experimental period. Thus, the increase of the complement activity (ACH50) in plasma of fish may help to identify and eliminate bacterial agents by phagocytosis. Therefore, the increased total protein levels in plasma of fish treated with watercress extract is accompanied by the increased levels of immune parameters which have a protein structure, such as globulins and total complements. Several authors reported an increase in complement activity following administration of different immunostimulants such as herbal derivatives (Jian and Wu, 2003; 2004; Christybapita *et al.*, 2007), sodium alginate (Bagni *et al.*, 2005; Cheng *et al.*, 2008), and vitamins C and E (Ortuno *et al.*, 1999; 2001).

Peroxidases are a large family of enzymes including myeloperoxidase, which plays an important role as a natural antibacterial agent in animal’s immune system (Clark and Klebanoff, 1975). Based on results, oral administration of watercress extract did not significantly affect peroxidase activity in plasma of fish when compared with control group at the end of experimental period.

Christybapita *et al*. (2007) recorded an increase in myeloperoxidase activity in tilapia fed with diets supplemented with different levels of aqueous extract of *Eclipta alba* for 1 week, whereas they didn’t reported any significant changes in myeloperoxidase activity after two or three weeks.

In the present study, the enhancement of total protein by using 1% of watercress extract supplementary food has been widely observed in fish. Since, there is a close relationship between the level of protein synthesis in liver tissue and plasma protein pools, total protein levels in plasma may be elevated due to the increased levels of protein synthesis in liver tissue of fish treated with watercress extract.

Banaee *et al*. (2011) reported that oral administration of some herbal medicine such as silymarin may improve protein synthesis in fish liver tissue. Consequently, significant increase of the total protein levels in plasma in treated fish is probably reflecting the increase of the protein synthesis in liver tissue. Similarly, the highest serum protein level was recorded in Nile tilapia fed yellow leader and Japanese honeysuckle (Ardó *et al.*, 2008), ginger, mistletoe and stinging nettle (Dügenci *et al.*, 2003). Proteins include albumin and globulin; some globulins are produced in the liver, while others are made by the immune system (Sandnes *et al.*, 1988). Globulin is made up of subunit of α1, α2, β, and γ globulins, which are considered as the source of almost all the immunologically active proteins in the blood (Jha *et al.*, 2007). Commonly, increases in the levels of plasma total protein, albumin and globulin in fish are thought to be associated with a stronger innate immune response (Wiegertjes *et al.*, 1996).

Although albumin did not increase in most of the treatment groups in the present study, globulin responded similarly to total protein, which certainly increased. Since albumin plays an important role in transport of some compounds such as drug in blood, minor increase albumin levels in plasma of experimental fish may help to transport of watercress extract in blood. Therefore, the increase of globulins in plasma of fish treated by 1% of watercress extract may indicate enhanced immune system of fish.

In conclusion, the results indicated that the use 1% of watercress extract as an immunostimulant in fish diets may have led to enhanced fish immunity. Since the watercress is rich in vitamin C, the increase of Hb and MCHC indicate that oral administration of the watercress extract dietary supplements can be effective in concentration of hemoglobin in erythrocyte of fish.

## References

[ref1] Abu-Zinadah O (2008). Effects of Watercress Oil on the Thermal and Chemical Burn Injuries in Rabbits. JKAU, Med. Sci.

[ref2] Aliyu R, Adebayo A.H, Gatsing D, Garba I. H (2007). The effects of ethanolic leaf extract of *Commiphora Africana* (Bureseraceae) on rat liver and kidney functions. J. Pharmacol. Toxicol.

[ref3] Ardó L, Yin G, Xu P, Váradi L, Szigeti G, Jeney Z, Jeney G (2008). Chinese herbs (*Astragalus membranaceus* and *Lonicera japonica*) and boron enhance the non-specific immune response of Nile tilapia (*Oreochromis niloticus*) and resistance against *Aeromonas hydrophila*. Aquaculture.

[ref4] Azizoglu A, Cengizler I (1996). An investigation on determination of some hematologic parameters in healthy *Oreochromis niloticus* (L.). Turk. J. Vet. Anim. Sci.

[ref5] Bagni M, Romano N, Finoia M.G, Abelli L, Scapigliati G, Tiscar P.G, Sarti M, Marino G (2005). Short and long-term effects of a dietary yeast ß-glucan (Macrogard) and alginic acid (Ergosan) preparation on immune response in sea bass (*Dicentrarchus labrax*). Fish Shellfish Immunol.

[ref6] Banaee M, Mirvagefei A.R, Rafei G.R, Amiri B.M (2008). Effect of sub-lethal Diazinon Concentrations on Blood Plasma Biochemistry. Int. J. Environ. Res.

[ref7] Banaee M, Sureda A, Mirvaghefi A.R, Rafei G.R (2011). Effects of long-term silymarin oral supplementation on the blood biochemical profile of rainbow trout (*Oncorhynchus mykiss*). Fish Physiol. Biochem.

[ref8] Bianchet M.A, Foster C, Faig M, Talalay P, Amzel L. M (1999). Structure and mechanism of cytosolic quinone reductases. Biochem. Soc. Trans.

[ref9] Biswas A.K, Kondaiah N, Anjaneyulu A.S.R, Mandal P. K (2010). Food safety concerns of pesticides, veterinary drug residues and mycotoxins in meat and meat products. Asian J. Anim. Sci.

[ref10] Brown B.A, Brown B.A (1988). Routine hematology procedures. Hematology, Principles and Procedures.

[ref11] Cheng A.C, Chen Y.Y, Chen J. C (2008). Dietary administration of sodium alginate and k-carrageenan enhances the innate immune response of brown-marbled grouper *Epinephelus fuscoguttatus* and its resistance against *Vibrio alginolyticus*. Vet. Immunol. Immunopathol.

[ref12] Christybapita D, Divyagnaneswari M, Michael R. D (2007). Oral administration of *Eclipta alba* leaf aqueous extract enhances the non-specific immune responses and disease resistance of *Oreochromis mossambicus*. Fish Shellfish Immunol.

[ref13] Chung F.L, Morse M.A, Eklind K.I, Lewis J (1992). Quantitation of human uptake of the anticarcinogen phenethyl isothiocyanate after a watercress meal. Cancer Epidemiol. Biomarkers Prev.

[ref14] Citarasu T, Sivaram V, Immanuel G, Rout N, Murugan V (2006). Influence of selected Indian immunostimulant herbs against white spot syndrome virus (WSSV) infection in black tiger shrimp, *Penaeus monodon* with reference to haematological, biochemical and immunological changes. Fish Shellfish Immunol.

[ref15] Clark R.A, Klebanoff S.J (1975). Neutrophil-mediated tumor cell cytotoxicity, Role of the peroxidase system. J. Exp. Med.

[ref16] Conaway C.C, Jiao D, Chung F. L (1996). Inhibition of rat liver cytochrome P450 isozymes by isothiocyanate and their conjugates, a structure activity relationship study. Carcinogenesis.

[ref17] Cuesta A, Vargas-Chacoff L, García-López A, Arjona F.J, Martínez-Rodríguez G, Meseguer J, Mancera J.M, Esteban M. A (2007). Effect of sex-steroid hormones, testosterone and estradiol, on humoral immune parameters of gilthead seabream. Fish Shellfish Immunol.

[ref18] Divyagnaneswari M, Christybapita D, Michael R. D (2007). Enhancement of nonspecific immunity and disease resistance in *Oreochromis mossambicus* by *Solanum trilobatum* leaf fractions. Fish Shellfish Immunol.

[ref19] Dügenci S.K, Arda N, Candan A (2003). Some medicinal plants as immunostimulant for fish. J. Ethnopharmacol.

[ref20] Goda Y, Hoshino K, Akiyama H, Ishikawa T, Abe Y, Nakamura T, Otsuka H, Takeda Y, Tanimura A, Toyoda M (1999). Constituents in Watercress, Inhibitors of Histamine Release from RBL-2H3 Cells Induced by Antigen Stimulation. Biol. Pharm. Bull.

[ref21] Hashemi S.R, Davoodi H (2012). Herbal plants as new immune-stimulator in poultry industry, a review. Asian J. Anim. Vet. Adv.

[ref22] Jha A.K, Pal A.K, Sahu N.P, Kumar S, Mukherjee S. C (2007). Haematoimmunological responses to dietary yest RNA, w-3 fatty acid and ß-carotene in *Catla catla* juveniles. Fish Shellfish Immunol.

[ref23] Ji S, Jeong G, Im G, Lee S, Yoo J, Takii K (2007). Dietary medicinal herbs improve growth performance, fatty acid utilization, and stress recovery of Japanese flounder. Fisheries Sci.

[ref24] Jian J, Wu Z (2003). Effects of traditional Chinese medicine on nonspecific immunity and disease resistance of large yellow croaker, *Pseudosciaena crocea* (Richardson). Aquaculture.

[ref25] Jian J, Wu Z (2004). Influence of traditional Chinese medicine on non specific immunity of Jian Carp (*Cyprinus carpio* var. Jian). Fish Shellfish Immunol.

[ref26] Kolawole S.O, Kolawole O.T, Akanji M. A (2011). Effects of aqueous extract of *Khaya* senegalensis stem bark on biochemical and hematological parameters in rats. J. Pharmacol. Toxicol.

[ref27] Lange S, Gudmundsdottir B.K, Magnadottir B (2001). Humoral immune parameters of cultured Atlantic halibut (*Hippoglossus hippoglossus* L.). Fish Shellfish Immunol.

[ref28] Martins M.L, Moraes F.R, Miyazaki D.M, Brum C.D, Onaka E.M, Fenerick J, Bozzo F. R (2002). Alternative treatment for *Anacanthorus penilabiatus* (Monogenea, Dactylogyridae) infection in cultivated pacu, *Piaractus mesopotamicus* (Osteichthyes, Characidae) in Brazil and its haematological effects. Parasit.

[ref29] Ndong D, Fall J (2011). The effect of garlic (*Allium sativum*) on growth and immune responses of hybrid tilapia (*Oreochromis niloticus* x *Oreochromis aureus*). J. Clin. Immunol. Immunopathol. Res.

[ref30] Obaroh I.O, Achionye-Nzeh G.C (2011). Effects of crude extract of *Azadirachta indica* leaves at controlling profile breeding in *Oreochromis niloticus* (Linnaeus, 1758). Asia J. Agric. Res.

[ref31] Ortuno J, Cuesta A, Esteban M.A, Meseguer J (2001). Effect of oral administration of high vitamin C and E dosages on the gilthead seabream (*Sparus aurata* L.) innate immune system. Vet. Immunol. Immunopathol.

[ref32] Ortuno J, Esteban M.A, Meseguer J (1999). Effect of high dietary intake of Vitamin C on nonspecific immune response of gilthead sea bream (*Sparus aurata* L.). Fish Shellfish Immunol.

[ref33] Palaniswamy U.R, McAvoy R.J, Bible B.B, Stuart J.D (2003). Ontogenic variations of ascrobic acid and phenathyl isothiocyanate concentration in watercress (Nasturtium officinale R.Br.) leaves. J. Agric. Food Chem.

[ref34] Rao Y.V, Chakrabarti R (2005). Stimulation of immunity in Indian major carp *Catla catla* with herbal feed ingredients. Fish Shellfish Immunol.

[ref35] Rao Y.V, Das B.K, Jyotyrmayee P, Chakrabarti R (2006). Effect of *Achyranthes aspera* on the immunity and survival of *Labeo rohita* infected with *Aeromonas hydrophila*. Fish Shellfish Immunol.

[ref36] Rooijakkers S.H, van Strijp J.A (2007). Bacterial complement evasion. Mol. Immunol.

[ref37] Rose P, Faulkner K, Williamson G, Mithen R (2000). 7-Methylsulfinylheptyl and 8- methylsulfinyloctyl isothiocyanates from watercress are potent inducers of phase II enzymes. Carcinogenesis.

[ref38] Sandnes K, Lie O, Waagbo R (1988). Normal ranges of some blood chemistry parameters in adult farmed Atlanic salmon, *Salmo salar*. J. Fish Biol.

[ref39] Sarder M.R, Thompson K.D, Penman D.J, McAndrew B. J (2001). Immune response of the Nile tilapia (*Oreochromis niloticus* L.) clones, 1. Non-specific responses. Dev. Comp. Immunol.

[ref40] Sarkar S.K, Sen U, Dhar M, Absar N, Islam M. K (2011). Evaluation of nutritive, antioxidant and mineral composition of two newly developed varieties of strawberry (*Fragaria ananassa*) and their antimicrobial activity and brine shrimp toxicity study. Asia J. Agric. Res.

[ref41] Shahrokhi N, Hadad M.K, Keshavarzi Z, Shabani M (2009). Effect of aqueous extract of watercress on glucose and lipid plasma in streptozotocin induced diabetic rats. Pak. J. Physiol.

[ref42] Shalaby A.M, Khattab Y.A, Abdel Rahman A. M (2006). Effects of garlic (*Allium sativum*) and chloramphenicol on growth performance, physiological parameters and survival of Nile tilapia (*Oreochromis niloticus*). J. Venom. Anim. Toxins incl. Trop. Dis.

[ref43] Sivaram V, Babu M.M, Immanuel G, Murugadass S, Citarasu T, Marian M. P (2004). Growth and Immune response of juvenile greasy groupers (*Epinephelus tauvina*) fed with herbal antibacterial active principle supplemented diets against *Vibrio harveyi* infections. Aquaculture.

[ref44] Sonnenbichler J, Goldberg M, Hane L, Madubunyi I, Vogl S, Zetl I (1986). Stimulatory Effect of Silibinin on DNA Synthesis in Partially Hepatectomized Rat Livers, Non-response in Hepatoma and Other Malign Cell Lines. Biochem. Pharmacol.

[ref45] Wallig M.A, Kingston S, Staack R, Jeffery E.H (1998). Induction of rat pancreatic glutathione -S- transferase and quinone reductase activities by a mixture of glucosinolate break down derivatives found in Brussel sprouts. Food Chem. Toxicol.

[ref46] Wiegertjes G.F, Stet R.J, Parmentier H.K, van Muiswinkel W. B (1996). Immunogenetics of disease resistance in fish; a comparable approach. Dev. Comp. Immunol.

[ref47] Yano T, Stolen J.S, Fletcher T.C, Anderson D.P, Hattari S.C, Rowley A.F (1992). Assay of hemolytic complement activity. In, Techniques in Fish Immunology.

[ref48] Yazdanparast R, Bahramikia S, Ardestani A (2008). *Nasturtium officinale* reduces oxidative stress and enhances antioxidant capacity in hypercholesterolemia rats. Chem. Biol. Interact.

[ref49] Yin G, Ardo L, Thompson KD, Adams A, Jeney Z, Jeney G (2009). Chinese herbs (*Astragalus radix* and *Ganoderma lucidum*) enhance immune response of carp, *Cyprinus carpio*, and protection against *Aeromonas hydrophila*. Fish Shellfish Immunol.

[ref50] Yin G, Jeney G, Racz T, Xu P, Jun X, Jeney Z (2006). Effect of two Chinese herbs (*Astragalus radix* and *Scutellaria radix*) on non-specific immune response of tilapia, *Oreochromis niloticus*. Aquaculture.

